# Molecular data for *Crenavolva* species (Gastropoda, Ovulidae) reveals the synonymy of *C. chiapponii*

**DOI:** 10.3897/zookeys.501.9144

**Published:** 2015-04-30

**Authors:** Bastian T. Reijnen

**Affiliations:** 1 Department of Marine Zoology, Naturalis Biodiversity Center, P.O. Box 9517, 2300 RA Leiden, The Netherlands

**Keywords:** *Acanthogorgia*, host association, molecular phylogeny, Octocorallia, 16S, COI

## Abstract

During fieldwork in Indonesia and Malaysia, eight lots containing 33 specimens belonging to the genus *Crenavolva* (Ovulidae) were collected. Species were initially identified as *Crenavolva aureola*, *Crenavolva chiapponii*, *Crenavolva striatula* and *Crenavolva trailli*, respectively. For *Crenavolva chiapponii* this is the second record. In contrast to the ecological data available from the original description of this species, it was found in shallow water on a gorgonian host coral, i.e. *Acanthogorgia* sp. A molecular analysis based on COI and 16S mtDNA markers, including sequence data obtained from GenBank, showed that *Crenavolva chiapponii* should be considered a junior synonym of *Crenavolva aureola* and that previously identified ovulid specimens are probably misidentified.

## Introduction

The nominal taxon Crenavolva was introduced as a subgenus by Cate (1973), together with the subgenera *Crenavolva*, *Cuspivolva* and *Serratovolva*. In the most recent overview regarding Ovulidae these three taxa are considered genera (Lorenz and Fehse 2009). At present 18 nominal species are recognized within *Crenavolva* (Rosenberg 2014), most of which are considered rare (Lorenz and Fehse 2009). These species are considered rare because few specimens have been collected, probably because they occur at depths greater than standard recreational diving depth of c. 30 m and/or are only known from a limited geographical area, usually just the type locality. This also accounts for *Crenavolva chiapponii* Lorenz & Fehse, 2009, which is only known from Balicasag Isl., Bohol, Philippines, where specimens were trawled from 70–120 m depth and, therefore, were considered rare and confined to deeper water (Lorenz and Fehse 2009). Like almost all other ovulids, species of *Crenavolva* are associated with octocoral hosts (Schiaparelli et al. 2005; Reijnen 2010) belonging to several families (e.g. Melithaeidae, Ellisellidae, Subergorgiidae and Plexauridae). However, the host species are usually not collected or are disregarded and therefore unknown, which is also the case for *Crenavolva chiapponii*.

Molecular data (16S and COI) obtained from *Crenavolva* was used by Meyer (2003) to root the phylogeny of the Cypraeidae. Later, the 16S sequence data were used by Schiaparelli et al. (2005) to produce the first molecular phylogenetic reconstruction of the Ovulidae, which included two *Crenavolva* species: Crenavolva cf. rosewateri (Cate, 1973) and *Crenavolva tokuoi* Azuma, 1989. In the present study, material of four additional nominal *Crenavolva* species, amongst other ovulids, have been used to reconstruct a phylogeny. The newly acquired molecular data are for *Crenavolva aureola* (Fehse, 2002), *Crenavolva chiapponii* Lorenz & Fehse, 2009, *Crenavolva striatula* (Sowerby I, 1828) (type species), and *Crenavolva trailli* (Adams, 1855). In addition to this phylogenetic reconstruction, data on host species and distributional records are given for this group of rarely recorded ovulid snails.

## Materials and methods

### Collection and identification

**Figure 1.**
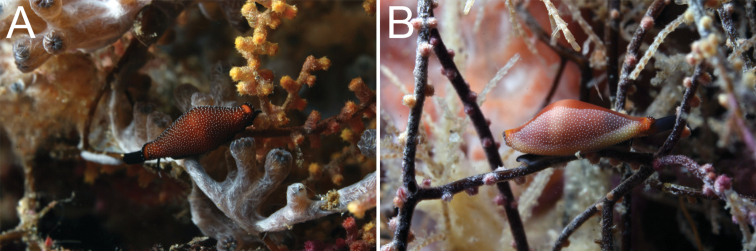
**A** *In situ* image of *Crenavolva aureola* (Fehse, 2002) (RMNH.MOL.164209) and **B** *Crenavolva chiapponii* Lorenz & Fehse, 2009 (RMNH.MOL.164211) on *Acanthogorgia* sp. at Halmahera, Indonesia at 21 m and 17 m depth respectively.

During fieldwork in Indonesia (Halmahera, Ternate; Sulawesi, Lembeh Strait) and Malaysia (Borneo, Semporna and Kudat) specimens of *Crenavolva* species were collected by SCUBA diving (Table 1). The snails and their octocoral hosts were photographed in situ (Fig. 1) whenever possible and subsequently fixed in 80% ethanol. The holotype of *Crenavolva chiapponii* was studied at the Muséum national d’Histoire naturelle (MNHN) in Paris. For the identification of the other ovulid species, Cate (1973), Fehse (2002b) and Lorenz and Fehse (2009) were used. For the identification of the host species, microscopy slides of their calcareous skeletal parts (sclerites) were made by dissolving the samples in a 4% solution of household bleach. The residual sclerites were rinsed with tap water followed by demineralised water before mounting on a slide or on a stub for Scanning Electron Microscopy (SEM). Stubs with sclerites were coated with Au/Pd before SEM images were made with a JEOL 6480 LV. Identification of the octocorals to genus level was based on Stiasny (1947) and Fabricius and Alderslade (2001).

**Table 1.** Specimens used in the analyses, including locality, host, and GenBank accession data.

Collection number	Species	Locality (Locality code)	Coordinates	Date collected	Host species	GenBank Accession number (16S; COI)	Reference
RMNH.MOL.164072	*Crenavolva aureola* (Fehse, 2002)	Malaysia, Semporna, Si Amil Island (SEM.16)	4°19'02.1"N; 118°52'30.7"E	4-12-2010	*Acanthogorgia* sp.	KP033143; KP033151	This publication
RMNH.MOL.164085	*Crenavolva aureola* (Fehse, 2002)	Indonesia, Halmahera, Tidore, N of Desa Rum (TER.18)	0°44'35.8"N; 127°23'06.3"E	4-11-2009	*Acanthogorgia* sp.	KP033144; KP033152	This publication
RMNH.MOL.164209	*Crenavolva aureola* (Fehse, 2002)	Indonesia, Halmahera, Tanjung Ratemu (S of river)(TER.21)	0°54'24.7"N; 127°29'17.7"E	5-11-2009	*Acanthogorgia* sp.	KP033148; KP033156	This publication
RMNH.MOL.164211	*Crenavolva chiapponii* Lorenz & Fehse, 2009	Indonesia, Halmahera, Tanjung Ratemu (S of river)(TER.27)	0°54'44.5"N; 127°29'09.9"E	8-11-2009	*Acanthogorgia* sp.	KP033157	This publication
RMNH.MOL.164217	*Crenavolva chiapponii* Lorenz & Fehse, 2009	Indonesia, Lembeh, Tanjung Kusukusu (LEM.31)	1°27'13.8"N; 125°14'13.0"E	16-2-2012	*Acanthogorgia* sp.	KP033149; KP033158	This publication
RMNH.MOL.164062	*Primovula rosewateri* (Cate, 1973)	Malaysia, Semporna, Kulapuan Island 2, N side (SEM.31)	4°32'07.4"N; 118°50'18.2"E	9-12-2010	*Paratelesto* sp.	KP033142; KP033150	This publication
RMNH.MOL.164186	*Crenavolva striatula* (Sowerby I, 1828)	Malaysia, Sabah, S Pulau Banggi, E Molleangan Besar Island, (TMP.37)	7°05'07.2"N; 117°03'33.8"E	19-9-2012	*Echinogorgia* sp.	KP033146; KP033154	This publication
RMNH.MOL.164144	*Crenavolva trailli* (Adams, 1855)	Malaysia, Sabah, Kalang, (TMP.41)	6°59'48.1"N; 117°03'13.4"E	18-9-2012	*Subergorgia* sp.	KP033145; KP033153	This publication
RMNH.MOL.164189	*Crenavolva trailli* (Adams, 1855)	Malaysia, Sabah, Kalang, (TMP.41)	6°59'48.1"N; 117°03'13.4"E	18-9-2012	*Paraplexaura* sp.	KP033147; KP033155	This publication
-	Crenavolva cf. rosewateri (Cate, 1973)	Philippines, Bohol, Balicasag Island	-	-	-	AY161394; AY161627	Meyer 2003
-	*Crenavolva tokuoi* Azuma, 1989	Philippines, Bohol, Balicasag Island	-	-	-	AY161390; AY161623	Meyer 2003
-	*Primovula beckeri* (Sowerby III, 1900)	Indonesia, Sulawesi	-	-	-	AJ868555; -	Schiaparelli et al. 2005
-	*Ovula ovum* (Linnaeus, 1758)	Indonesia, Sulawesi, Spermonde Archipelago	-	-	-	AY161399; AY161632	Meyer 2003

### Barcoding Ovulidae

Specimens were barcoded for the COI barcoding region and for additional phylogenetic research also for the 16S marker. Tissue samples obtained from the foot and/or mantle were extracted with the Machery-Nagel DNA extraction kit on a KingFisher Flex. The standard COI barcoding primers by Folmer et al. (1994) and the Palumbi (1996) 16S primers were used. PCR amplification was performed on a C1000 Touch Thermal Cycler (Bio-RAD). Sequencing of the PCR products was performed at Macrogen Europe on an ABI 3730xl Automated Sequencer. Sequences were edited in Sequencher 4.10.1 and aligned with GUIDANCE (Penn et al. 2010) using the MAFFT algorithm (Katoh et al. 2005). Selecting an evolutionary model was done with jModeltest based on the Akaike Information Criterion score. MEGA 6.0.6 (Tamura et al. 2013) was used to perform Maximum Likelihood (ML) and Maximum Parsimony (MP) analyses and to calculate p-distances. Bayesian analyses were performed in MrBayes 3.2.0 (Ronquist and Huelsenbeck 2003). MrBayes was run for 4,000,000 generations with six chains. Data were sampled every 100 generations. Sequence data for *Ovula ovum* (Linnaeus, 1758) from GenBank was used as an outgroup. GenBank data for Crenavolva cf. rosewateri (Cate, 1973), *Crenavolva tokuoi* Azuma, 1989 and *Primovula beckeri* (Sowerby III, 1900) was also included in the phylogenetic analyses.

## Results

### Collecting and morphology

**Figure 2.**
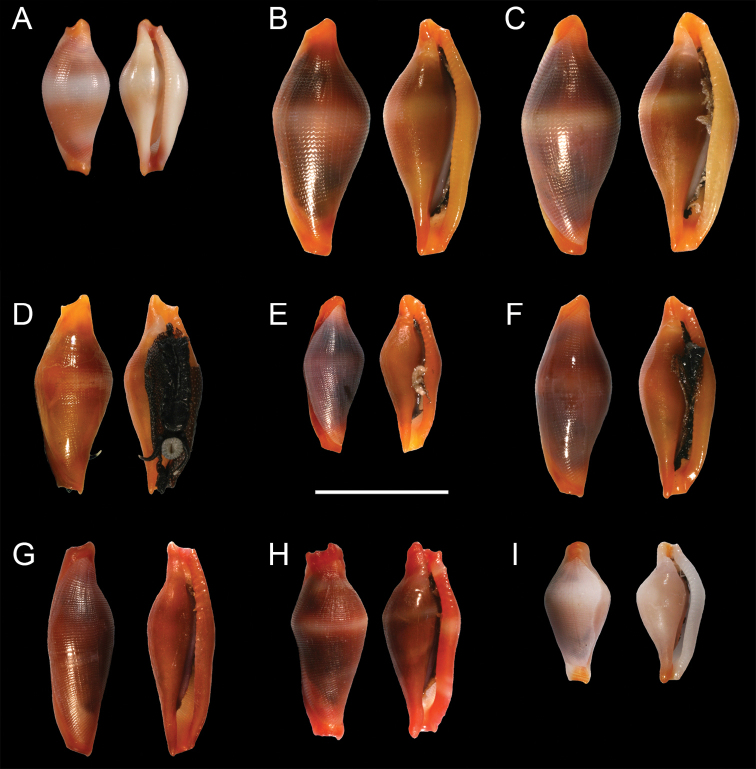
Dorsal and ventral views of shells. **A** Holotype of *Crenavolva chiapponii* Lorenz & Fehse, 2009 (MNHN 21244) **B** *Crenavolva chiapponii* Lorenz & Fehse, 2009 (RMNH.MOL.164211) **C** *Crenavolva chiapponii* Lorenz & Fehse, 2009 (RMNH.MOL.164217) **D** *Crenavolva aureola* (Fehse, 2002) (RMNH.MOL.164085) **E** *Crenavolva aureola* (Fehse, 2002) (RMNH.MOL.164072) **F** *Crenavolva aureola* (Fehse, 2002) (RMNH.MOL.164209) **G** *Crenavolva trailli* (Adams, 1855) (RMNH.MOL.164144) **H** *Crenavolva striatula* (Sowerby I, 1828) (RMNH.MOL.164186) **I** *Primovula rosewateri* (Cate, 1973) (RMNH.MOL.164062). Scale bars: 5 mm.

Eight lots, containing 33 specimens representing four nominal *Crenavolva* species (*Crenavolva aureola*, *Crenavolva chiapponii*, *Crenavolva striatula* and *Crenavolva trailli*) were collected in Indonesia and Malaysia (Table 1; Fig. 2). For *Crenavolva chiapponii* this is the first record from shallow water. The specimens were assigned to these nominal species based on shell shape (rhomboid, inflated or slender) and the colour bands on the dorsum, which in case of *Crenavolva striatula* were also present on the labrum. For *Crenavolva aureola* and *Crenavolva chiapponii* the absence or presence of a white dorsal band on the shell is allegedly the most obvious character to distinguish the species. After examination of the illustrations presented by Lorenz and Fehse (2009) and the newly collected material, minor morphological differences (strongly or weakly pronounced dentation, keeling angle, strongly or weakly produced funiculum, position of the widest part of the shell) do not clearly separate between *Crenavolva aureola* and *Crenavolva chiapponii* and can be considered morphological variation in a single species. The soft tissue colouration of both *Crenavolva aureola* and *Crenavolva chiapponii* is very similar (e.g. Fig. 1; Lorenz and Fehse 2009: A106, A107 p. 527). Both have a semi-transparent mantle which is entirely covered with small, irregularly placed, white dots, and both have a completely black or white foot, black tentacles with white tips, and a black siphon.

### Molecular data

**Figure 3.**
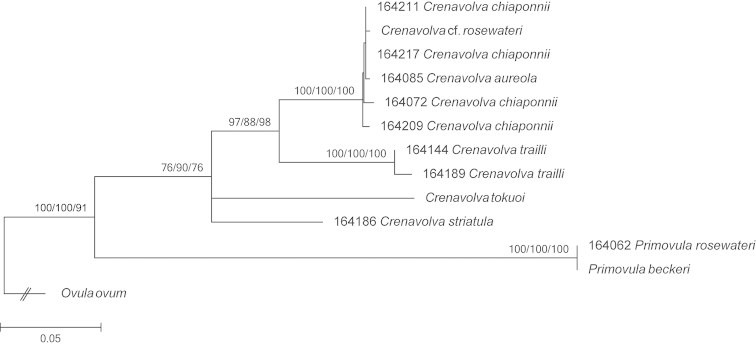
Maximum Likelihood cladogram with support values for the ML/MP/BP analyses. Numbers preceding the species names represent RMNH.MOL. collection numbers of Naturalis Biodiversity Center; species names without numbers are obtained from GenBank for which additional data can be found in Table 1.

Nine specimens representing five species were sequenced for COI and 16S. For one sample of *Crenavolva chiapponii* (RMNH.MOL.164211) the 16S marker could not be amplified. Sequences were concatenated and aligned (GUIDANCE alignment score: 0.965034) which resulted in an alignment length of 1080 base pairs per specimen including indels. Sequences obtained from GenBank are slightly shorter (~40 base pairs), these missing base pairs were coded as ‘missing data’. The program jModeltest yielded in HKY+G as most optimal evolutionary model. This evolutionary model was implemented in the Bayesian and ML analysis. The results from the different phylogenetic reconstructions were congruent, therefore only the ML tree is shown (Fig. 3).

In the phylogenetic reconstructions, specimens of *Crenavolva striatula* and *Crenavolva tokuoi* form an unresolved trichotomy with the other *Crenavolva* specimens. The two *Primovula* species cluster together and are well-supported sister species to all the *Crenavolva* species (with *Crenavolva striatula* as type species for the genus). This implies that the *Crenavolva* species used herein form a monophyletic group. The clustering of two *Crenavolva trailli* specimens is highly supported. Another well-supported clade holds three nominal species: *Crenavolva aureola*, *Crenavolva chiapponii* and Crenavolva cf. rosewateri. The pairwise p-distances between these three species are very low (16S: 0.2%; COI: 0.7%; concatenated: 0.9%). In contrast, the sequence divergence between *Crenavolva trailli* and the *Crenavolva chiapponii* / *Crenavolva aureola* clade is almost ten times larger (16S: 5.2%; COI: 8.7%; concatenated: 8.2%). The sequence divergence between the two *Crenavolva trailli* specimens (16S: 0.6%; COI: 0.8%; concatenated: 0.8%) is almost equal to that between *Crenavolva aureola* and *Crenavolva chiapponii*. With the help of the Automatic Barcode Gap Discovery tool (ABGD) (Puillandre et al. 2011), the data were analysed to identify the MOTU’s within the dataset. The results of this analysis showed that the barcode gap to identify the different species is 5–6% sequence divergence. This resulted in five groups containing the following species: 1, *Crenavolva aureola*, *Crenavolva chiapponii*, Crenavolva cf. rosewateri; 2, *Crenavolva trailli*; 3, *Crenavolva tokuoi*; 4, *Crenavolva striatula*; 5, *Primovula rosewateri*. One of the samples obtained from GenBank, viz. Crenavolva cf. rosewateri (= Primovula cf. rosewateri), clusters surprisingly within the clade containing *Crenavolva aureola* and *Crenavolva chiapponii* and not with the other *Primovula rosewateri* specimen. Instead, *Primovula beckeri* proves to be identical to the newly sequenced specimen of *Primovula rosewateri* from Malaysia.

### Octocoral hosts

Almost all Ovulidae species are associated with Octocorallia hosts. By examining the sclerites and the habitus of the host corals, several new host species for ovulids of the genus *Crenavolv* a could be identified. An overview of previously identified host species and new records is provided in Table 2. Some of the former host identifications were published with obsolete generic names, and therefore their names in the current literature are also provided. Before *Crenavolva chiapponii* was synonymised, *Acanthogorgia* would have been a new host record. Yet, Reijnen (2010) already recorded *Acanthogorgia* sp. as a host for *Crenavolva aureola* and therefore it is not a new host record. Morphologically at least two different species of *Acanthogorgia* could be distinguished but these could not be identified since a revision of the family Acanthogorgiidae is lacking.

**Table 2.** Literature overview of the octocoral hosts of selected *Crenavolva* species including new records. Updated names of the octocoral hosts are provided between parentheses.

Ovulid species	Host genera	Reference
*Crenavolva aureola*	*Euplexaura*; *Astromuricea* (= *Echinogorgia*); *Acanthogorgia*	Lorenz and Fehse 2009; Reijnen 2010
*Crenavolva chiapponii* (= *Crenavolva aureola*)	*Acanthogorgia*	this publication; Reijnen 2010
*Crenavolva striatula*	*Ellisella*; *Euplexaura*; *Echinogorgia*	Lorenz and Fehse 2009; Yamamoto 1973; Cumming 1997; Mase 1989;
*Crenavolva trailli*	*Echinogorgia*; *Anthoplexaura* (= *Astrogorgia*); *Plexauroides* (= *Echinogorgia*); *Euplexaura*; *Subergorgia*	Goh et al. 1999; Mase 1989
*Primovula rosewateri*	*Subergorgia*; *Dendronephthya*; *Stereonephthya*; *Paratelesto*	Goh et al. 1999; Lorenz and Fehse 2009; this publication
*Primovula beckeri*	*Acanthogorgia*; *Acabaria* (= *Melithaea*); *Unicella* [sic] (= *Eunicella*); *Lophogorgia* (= *Leptogorgia*)	Schiaparelli et al. 2005; Lorenz and Fehse 2009

Furthermore, examination of the ovulid species and their octocoral hosts revealed that in two instances individuals formerly identified as *Crenavolva chiapponii* and *Crenavolva aureola* would have co-occurred on the same host coral, in both cases *Acanthogorgia* sp.

## Discussion

Based on the molecular data and morphological observations listed above, *Crenavolva chiapponii* is considered a junior synonym of *Crenavolva aureola*. The systematic account is therefore as follows:

### Systematic part

#### Family Ovulidae Fleming, 1822 Genus *Crenavolva* Cate, 1973

##### *Crenavolva aureola* (Fehse, 2002)

*Primovula aureola* Fehse 2002: 37, pl. 1, fig. 1

*Delonovolva formosa*. — Gosliner et al. 1996: 136, fig. 469. Not *Delonovolva formosa* (Sowerby II in Adams and Reeve 1848) [= *Cuspivolva formosa* (Sowerby II in Adams and Reeve 1848)]

*Primovula* sp. — Coleman 2003: 51, fig. (Ovul: 121).

*Crenavolva chiapponii*Lorenz and Fehse 2009: 69, pl. 74, fig. 7–11.

The occurrence of *Crenavolva chiapponii* (= *Crenavolva aureola*) on Indonesian shallow water coral reefs would have represented new distribution records, both geographically and bathymetrically, before it was synonymised. However *Crenavolva chiapponii* proved to be a junior synonym of *Crenavolva aureola* and the new distribution records fall within the distribution range already known for *Crenavolva aureola*. Apparently, the dorsal white band and the minor morphological differences in shell shape are not indicative of species-level differences between *Crenavolva aureola* and *Crenavolva chiapponii*.

#### Molecular data

The species *Primovula rosewateri* was previously placed in the genus *Crenavolva* by Cate (1973) but Fehse (2002a) moved it to *Primovula*, primarily based on the triangular shape of the funiculum. The results of the molecular analyses (Fig. 3) support this decision. There is great genetic similarity between Crenavolva cf. rosewateri (= Primovula cf. rosewateri) obtained from GenBank, and *Crenavolva aureola*. However, the specimen from GenBank was collected from Balicasag Island, near Bohol, Philippines, which is the type locality of *Crenavolva chiapponii*. This location is approximately 85 km from Mactan Island of Cebu, Philippines which is the type locality of *Crenavolva aureola*. It is not unlikely that the so-called Crenavolva cf. rosewateri from GenBank (AY161394 (16S), AY161627 (COI)) was misidentified and actually represents *Crenavolva aureola*. Moreover, the newly sequenced specimen of *Primovula rosewateri* from Malaysia convincingly clusters with *Primovula beckeri*. According to Lorenz and Fehse 2009, *Primovula beckeri* has an E African distribution and was originally described from South Africa. The specimen obtained from GenBank is from Sulawesi, Indonesia (Schiaparelli et al. 2005). It is therefore unlikely that this sequence represents *Primovula beckeri* but instead is the quite similar species from the central Indo-Pacific, *Primovula rosewateri*.

#### Host species and distribution records

The ranges of the presently discussed species all fit within the Coral Triangle (see Hoeksema 2007) and depend on the ranges of their host species. Species of the genus *Acanthogorgia* are not unique hosts for just *Crenavolva* spp. Reijnen (2010) already mentioned *Acanthogorgia* spp. as a host for *Dentiovula eizoi* Cate & Azuma, 1973 (in Cate 1973) and *Dentiovula colobica* (Azuma & Cate, 1971). *Acanthogorgia* species and their ovulid associates are both known to occur from shallow to deep water in the Coral Triangle. In an overview of the Acanthogorgiidae by Stiasny (1947) the deepest record for an *Acanthogorgia* species is 4239 m, collected SE of Seram, Indonesia (*Acalycigorgia densiflora* = *Acanthogorgia densiflora* (Kükenthal & Gorzawsky, 1908). Nevertheless, Stiasny (1947) doubts the identification and compared it to congeneric species which are found in waters not exceeding 400 m depth. As a result Stiasny (1947) doubts the entire record. Therefore the deepest reliable record for an *Acanthogorgia* species in the Malayan Archipelago is 1254 m for *Acanthogorgia multispina* (Kükenthal & Gorzawsky, 1908). The deepest record for *Crenavolva* species is from approximately 1000 m, which is the deepest record for any ovulid species found to date (Lorenz and Fehse 2009).
